# Angiopoietin-2 in Adults with Congenital Heart Disease and Heart Failure

**DOI:** 10.1371/journal.pone.0066861

**Published:** 2013-06-24

**Authors:** Alexander Lukasz, Gernot Beutel, Philipp Kümpers, Agnieszka Denecke, Mechthild Westhoff-Bleck, Bernhard Schieffer, Johann Bauersachs, Jan T. Kielstein, Oktay Tutarel

**Affiliations:** 1 Department of Nephrology and Hypertension, Hannover Medical School, Hannover, Germany; 2 Department of Medicine D, Division of General Internal Medicine, Nephrology, and Rheumatology, University Hospital Muenster, Muenster, Germany; 3 Department of Hematology and Oncology, Hannover Medical School, Hannover, Germany; 4 Department of Cardiology and Angiology, Hannover Medical School, Hannover, Germany; University of Otago, New Zealand

## Abstract

**Background:**

Chronic heart failure is an important cause for morbidity and mortality in adults with congenital heart disease (ACHD). While NT-proBNP is an established biomarker for heart failure of non-congenital origin, its application in ACHD has limitations. The angiogenic factors Angiopoietin-1 and -2 (Ang-1, Ang-2), vascular endothelial growth factor (VEGF), and soluble receptor tyrosine kinase of the Tie family (sTie2) correlate with disease severity in heart failure of non-congenital origin. Their role in ACHD has not been studied.

**Methods:**

In 91 patients Ang-2 and NT-proBNP were measured and related to New York Heart Association class, systemic ventricular function and parameters of cardiopulmonary exercise testing. Ang-1, VEGF, and sTie2 were also measured.

**Results:**

Ang-2 correlates with NYHA class and ventricular dysfunction comparable to NT-proBNP. Further, Ang-2 showed a good correlation with parameters of cardiopulmonary exercise testing. Both, Ang-2 and NT-proBNP identified patients with severely limited cardiopulmonary exercise capacity. Additionally, Ang-2 is elevated in patients with a single ventricle physiology in contrast to NT-proBNP. VEGF, Ang-1, and sTie2 were not correlated with any clinical parameter.

**Conclusion:**

The performance of Ang-2 as a biomarker for heart failure in ACHD is comparable to NT-proBNP. Its significant elevation in patients with single ventricle physiology indicates potential in this patient group and warrants further studies.

## Introduction

Chronic heart failure (CHF) is an important cause for morbidity and mortality in adults with congenital heart disease (ACHD) [1]. Heart failure symptoms may not always correlate with objective measures like systemic ventricular function or parameters of cardiopulmonary exercise testing [2], [3]. The rarity of individual malformations and the complex anatomy and physiology make assessing the cardiac function difficult [4]. Therefore, the prevalence of heart failure in these patients is underappreciated [5], [6]. A simple investigation like a blood test to detect early stages of heart failure and predict those at risk of deterioration would be valuable [4]. B-type natriuretic peptide (BNP) and N-terminal pro-BNP (NT-proBNP) are established biomarkers for diagnosis and management of heart failure due to acquired heart disease [7]. Unfortunately, the clinical use of those markers in adults with congenital heart disease is limited [8], [9]. Therefore diagnosis and treatment monitoring is frequently based on cardiopulmonary exercise testing [4], [6], [10], [11], which is time consuming and not feasible in special patient groups.

Endothelial dysfunction is one of the hallmarks in patients with chronic heart failure of non-congenital origin, and has also already been described in some forms of congenital heart disease [12],[13]. Recently we provided evidence for the promising role of circulating levels of asymmetrical dimethylarginine (ADMA), the most potent endogenous nitrix oxid synthase (NOS) inhibitor, for the diagnosis of heart failure in patients with ACHD [14]. Angiopoietins as growth factors of angiogenesis also play a role in endothelial dysfunction. Angiopoietin-1 (Ang-1) and Angiopoietin-2 (Ang-2) are antagonistic ligands of the Tie2 receptor, which is the second vascular specific receptor tyrosine kinase (the first being the vascular endothelial growth factor (VEGF)/VEGF receptor). The Ang/Tie2 ligand receptor system is a non-redundant gatekeeper of endothelial activation and controls the endothelial phenotype during angiogenesis and inflammation [15],[16]. Ang-1 is continuously produced and released by pericytes. Binding of Ang-1 to Tie2 enhances vascular integrity, prevents vascular leakage and suppresses inflammatory gene expression [17],[18]. In contrast, Ang-2 competitively inhibits binding of Ang-1 to Tie2 and thereby disrupts protective Ang-1 signalling leading to loss of vessel integrity, vascular leakage and expression of inflammatory genes [16],[19]. Ang-2 is stored in the endothelium and rapidly released upon different activators e.g. hypoxemia [20]. Recently published studies report a significant increase of the soluble Tie2 receptor, Ang-2 and VEGF in patients with CHF due to acquired heart disease when compared with healthy controls [21],[22]. Serum Ang-2 correlates with an impaired exercise capacity and reduced ventilatory capacity in CHF patients [22]. The role of these circulating endothelial factors in ACHD has not been studied before. Therefore, the aim of this study was to elucidate the potential diagnostic value of Ang-1, Ang-2, soluble Tie2 and VEGF for heart failure in adults with congenital heart disease.

## Materials and Methods

### Ethics Statement

The study was approved by the local Ethics Committee of Hannover Medical School, Germany. All patients gave written informed consent.

The patients were recruited during a routine outpatient visit at the Adult Congenital Heart Disease Clinic of the Hannover Medical School. All patients in whom a venous blood sampling was feasible were eligible for this study.

A clinical workup including medical history, physical examination, 12-lead electrocardiogram, transthoracic echocardiography, and cardiopulmonary exercise testing was performed.

The severity of the congenital heart defect was graded according to complexity as proposed by guidelines [23]. The patients were further classified according to their symptoms of heart failure using the New York Heart Association (NYHA) functional classification which is based on patient’s symptoms and the limitations to normal physical activities [7].

### Laboratory Methods

Blood samples for measurement of plasma Ang-1, Ang-2, VEGF, sTie2 and NT-proBNP, and routine biochemistry were drawn. Blood samples were immediately cooled on ice, centrifuged at 1,500 g and 4°C for 10 min. Supernatants were stored in 1 ml aliquots at –80°C until further use. Plasma concentrations of Ang-1 and Ang-2 were measured by an in-house immunoluminometric assay as was previously reported in detail [24]. In brief the assays had detection limits of 0.12 ng/mL (Ang-1) and 0.2 ng/mL (Ang-2). Inter- and intra-assay imprecision was ≤8.8 and 3.7% for Ang-1 and was ≤4.6 and 5.2% for Ang-2, respectively. Plasma VEGF (biologically active VEGF-A121 and VEGF-A165) and sTie2 were measured using commercially available sandwich ELISA kits (R&D Systems, Minneapolis, MN, USA) according to the manufacturer’s instructions. All other measurements were done with routine laboratory tests using certified assay methods.

### Echocardiography

A standard 2D-Doppler transthoracic echocardiogram was performed according to the recommendations for the assessment of ventricular function and valvular heart disease issued by the American Society of Echocardiography [25]. Systemic ventricular systolic function was assessed qualitatively (i.e. normal, moderately or severely impaired).

### Cardiopulmonary Exercise Studies

Cardiopulmonary exercise studies were performed on a bicycle in sitting position, starting with 25 W, increasing further 25 W every 2 min. All patients exercised to the end of their tolerance. A 12-lead ECG was recorded throughout the exercise test to determine heart rate and heart rate response. Systolic blood pressure and blood pressure response, as well as work rate (W/kg) were measured. Ventilation, oxygen uptake (VO_2_), and carbon dioxide production (VCO_2_), were measured continuously by a breath-by-breath method. Subjects breathed through a fitted mask and a hot-wire anemometer (Oxycon Delta, Jäger, Hoechberg, Germany) measuring inspired and expired flow continuously.

### Statistical Analysis

Continuous data are presented as mean ± standard deviation. Categorical data are presented as counts and proportions. Between group comparisons were examined using Student’s *t* test for continuous and Mann-Whitney-U test for categorical variables. One-way ANOVA was used if more than two groups were compared. The least significant difference (LSD) method was used as a post hoc-test.

For the parameters of cardiopulmonary exercise testing cut off values representing patients with limitations of their cardiopulmonary exercise capacity were defined as previously described [14]: peak oxygen uptake (peak VO_2_) <20 ml/min/kg, ventilatory equivalent for carbon dioxide (EQCO_2_) >34, ventilatory equivalent for oxygen (EQO_2_) >34, oxygen pulse if female <9 ml/heartbeat, if male <12 ml/heartbeat. A group of patients that was severely affected was defined by a peakVO_2_<20 ml/min/kg or an EQCO_2_>34 or a combination of both. The cut-offs were chosen according to published data and clinical experience [2],[3],[26].

Receiver-operating characteristic curves (ROC curve) for these parameters were drawn and the areas under the curves calculated. All tests were two-sided and significance was accepted at p<0.05. Data analysis was performed using SPSS (SPSS Inc., Chicago, IL, USA). Figures were prepared using GraphPad Prism (GraphPad Prism Software Inc., San Diego, CA, USA).

## Results

Ninety-one patients were included in this cross-sectional study. Ang-1, VEGF and Tie2 could only be analyzed in 80 patients. **Table 1** and **Table 2** show the clinical characteristics of the study population. Cardiopulmonary exercise testing was performed in 70 patients. Five of these performed a submaximal exercise test (respiratory exchange ratio <1.05) and were excluded from analysis. Nine patients were cyanotic. Out of these three had an exercise test. The values for EQCO_2_ were not used in these patients since EQCO_2_ is not a marker of prognosis in cyanotic patients.

**Table 1:** Clinical characteristics of study population.

**Age** (yrs)	30.4±10.7
**Sex**	
female	37 (40.7)
male	54 (59.3)
**BMI** (kg/m^2^)	23.5±4.2
**Complexity of congenital heart disease**	
simple	18 (19.8)
moderate	36 (39.6)
severe	37 (40.7)
**Systemic ventricle**	
left	65 (71.4)
right	12 (13.2)
single ventricle	14 (15.4)
**Systemic ventricular function**	
normal	52 (57.1)
moderately impaired	32 (35.2)
severely impaired	7 (7.7)
**NYHA class**	
NYHA I	55 (60.4)
NYHA II	21 (23.1)
NYHA III	15 (16.5)

Data are expressed as mean±SD or as counts (percentage).

**Table 2:** Type of congenital heart defect grouped according to ventricular function.

		Ventricular function		
Congenital heart defect	All	Normal	Moderately impaired	Severely impaired
TGA and CCTGA	11 (12.1)	2 (18.2)	5 (45.5)	4 (36.4)
Tetralogy of Fallot	12 (13.2)	6 (50)	6 (50)	0
Coarctation of the aorta	10 (11.0)	9 (90)	1 (10)	0
ASD or VSD	11 (12.1)	9 (81.8)	2 (18.2)	0
AVSD	5 (5.5)	5 (100)	0	0
Marfan syndrome	7 (7.7)	3 (42.9)	4 (57.1)	0
Congenital AS or PS	11 (12.1)	9 (81.8)	2 (18.2)	0
Single ventricle physiology	13 (14.3)	4 (30.8)	6 (46.2)	3 (23.1)
Miscellaneous	11 (12.1)	5 (45.5)	6 (54.5)	0

Data are expressed as counts (percentage); TGA = transposition of the great arteries; CCTGA = congenital corrected transposition of the great arteries; ASD = atrial septal defect; VSD = ventricular septal defect; AVSD = atrioventricular septal defect; AS = aortic valve stenosis; PS = pulmonary valve stenosis; miscellaneous: Ebstein’s anomaly, subaortic stenosis, supravalvular aortic stenosis, pulmonary atresia, Eisenmenger.

### Ventricular Function

**Figure 1:**
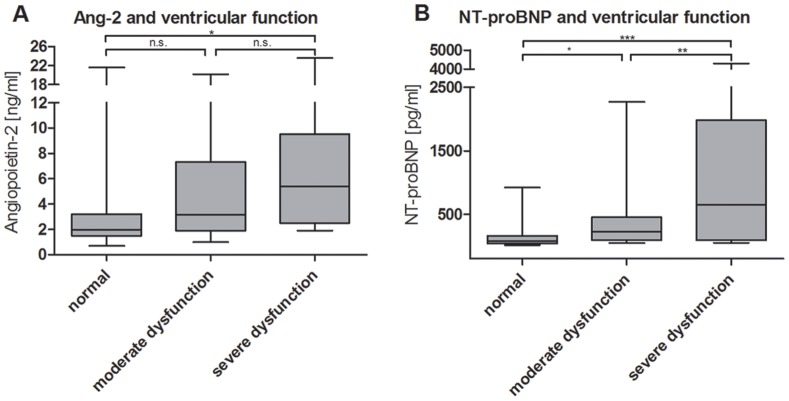
Levels of biomarkers in comparison between normal, moderate and severe impairment of ventricular function. Ang-2 (A) and NT-proBNP (B) (*p<0.05, **p<0.001, ***p<0.0001).

Ang-1, VEGF and Tie2 were not statistically different in patients with normal ventricular function compared to patients with moderate or severe ventricular dysfunction. (**Table 3**). Ang-2 did reach a statistically significant difference in patients with normal ventricular function compared to patients with severe ventricular dysfunction (3.53±4.19 ng/ml vs. 7.48±7.57 ng/ml, p<0.05), but not to those with moderate ventricular dysfunction (5.3±5.18, p = 0.11). There was also no significant difference between the last two. (**Table 3** and **Figure 1A**).

**Table 3:** Ang-1, Ang-2, VEGF, Tie2 and NT-proBNP levels according to NYHA class, ventricular function and systemic ventricle physiology.

	Ang-1 (ng/ml)	Ang-2 (ng/ml	VEGF (pg/ml)	Tie2 (ng/ml)	NT-proBNP (pg/ml)
***NYHA Class***					
I	5.8±3.8	2.5±1.6	111.4±62.5	19.9±3.3	128±200
II	6.5±5.7	6.8±6.6^a^	119.2±114.4	23.1±5.7	432±517^a^
III	4.5±3.2	8.2±6.9^b^	156.7±142.7	21.0±3.9	796±1081b,c
***Ventricular function***					
normal	5.1±3.2	3.5±4.2	117.7±84.8	20.5±4.2	142±177
moderately impaired	6.7±5.5	5.3±5.1	128.6±111.1	21.0±4.1	394±477^d^
severely impaired	5.7±3.8	7.5±7.6^e^	122.2±110.8	23.2±4.4	1156±1540^f^
***Systemic ventricle***					
right	7.5±7.3	4.9±5.2	117±85.5	21.6±4.2	642±758
left	5.6±3.4	2.9±2.8	116.9±81.6	20.1±3.7	180±250^g^
single	4.6±3.0	11.2±6.9^h^	144.5±145.9	23.1±5.4	617±1091^i^

Values are expressed as mean ± SD;

a p<0.01 to NYHA I - Ang-2 and p<0.05 for NT-proBNP;

b p<0.001 to NYHA I - Ang-2 and NT-proBNP;

c p<0.05 for NT-proBNP and NYHA II;

d p<0.01 vs. normal ventricular function;

e p<0.05 vs. normal ventricular function;

f p<0.01 vs. moderate ventricular dysfunction and p<0.001 vs. normal ventricular function;

g p<0.01 vs. systemic right ventricle;

h p<0.001 vs. systemic left and right ventricle;

i p<0.01 vs. systemic left ventricle.

NT-proBNP reached a statistically significant difference in patients with normal ventricular function compared to those with severe ventricular dysfunction (142±177 pg/ml vs. 1156±1540 pg/ml, p<0.0001) and between patients with normal ventricular function and moderate ventricular dysfunction (142±177 pg/ml vs. 394±477 pg/ml, p<0.05) as well as between patients with moderate and severe ventricular dysfunction (394±477 pg/ml vs. 1156±1540 pg/ml, p<0.001). (**Table 3** and **Figure 1B**).

### NYHA Class

**Figure 2:**
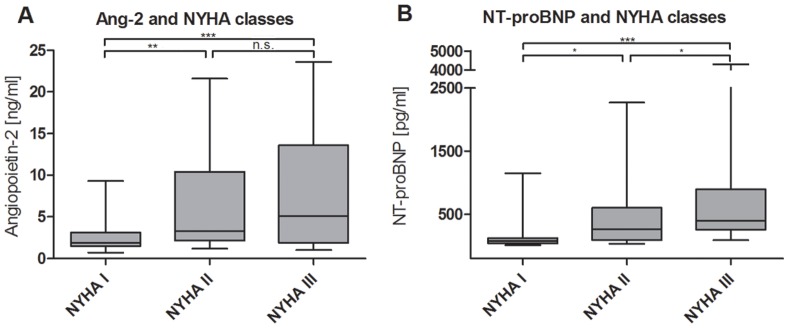
Levels of biomarkers in comparison between NYHA classes. Ang-2 (A) and NT-proBNP (B) (*p<0.05, **p<0.001, ***p<0.0001).

Ang-2 differentiated between patients in NYHA class I (2.52±1.6 ng/ml) and NYHA class II (6.83±6.56 ng/ml, p<0.0001), as well as those in NYHA class III (8.23±6.88 ng/ml, p<0.0001), but not between patients in NYHA class II and III (p = 0.34). (**Table 3** and **Figure** **2A**) The results for Ang-2 as a function of NYHA class in each ventricular function subset can be found in **Table 4**. The highest values were found in the subgroup of patients in NYHA class III and severe ventricular function, while patients in NYHA class I and normal ventricular function had the lowest values. It seems that Ang-2 could mark NYHA class within a given ventricular function better than marking ventricular function within a given NYHA class, but the sample size in each group is too small to provide conclusive answers.

**Table 4:** Ang-2 as a function of NYHA class in each ventricular function subset.

	NYHA I		NYHA II		NYHA III			
Ventricular function	n	Ang-2	n	Ang-2		n	Ang-2	
*normal*	40	2.2±1.4	7	8.1±7.7^a^		5	7.6±6.3^b^	
*moderately impaired*	15	3.3±1.8^c^	11	6.6±6.9^a^		6	7.8±6.3a,d	
*severely impaired*	0	n/a	3	4.7±1.9		4	9.6±9.9a,d	

Values are expressed as mean ± SD;

a p<0.01 to NYHA I – normal;

b p<0.05 to NYHA I – normal;

c p<0.05 to NYHA II – normal;

d p<0.05 to NYHA I – moderate. All other comparisons were not significant.

NT-proBNP was significantly different in patients in NYHA class I (128±202 pg/ml) compared to patients in NYHA class III (796±1081 pg/ml, p<0.0001) and to patients in NYHA class II (432±517 pg/ml, p<0.05) as well as between the last two (p<0.05). (**Table 3** and **Figure 2B**). Ang-1, VEGF and Tie2 were not different between NYHA classes. (**Table 3**).

### Cardiopulmonary Exercise Testing

Peak VO_2_ was significantly higher in patients in NYHA class I (28.6±7.4 ml/min/kg) vs. patients in NYHA class II (23.3±4.5 ml/min/kg, p = 0.01) and patients in NYHA class III (14.1±5.2 ml/min/kg, p<0.001) and also in comparison between NYHA class II and III (p = 0.001). Patients with a normal ventricular function had a significantly higher peak VO_2_ (28.5±7.6 ml/min/kg) compared to patients with a moderate (20.6±7.2 ml/min/kg, p<0.001) or severe ventricular dysfunction (21.0±8.4 ml/min/kg, p = 0.018). There was no significant difference between the last two (p = 0.917).

Ang-2 was elevated in patients with limited cardiopulmonary exercise compared to their peers. Significant differences were observed for EQO_2_ (p = 0.04), and oxygen pulse (p = 0.03), but not for peak VO_2_ (p = 0.23) and EQCO_2_ (p = 0.11). NT-proBNP was not elevated in patients with limited cardiopulmonary exercise capacity. There was no significant difference for peak VO_2_ (p = 0.16), EQCO_2_ (p = 0.30), EQO_2_ (p = 0.12), and oxygen pulse (p = 0.05). (**Table 5**).

**Table 5:** Association of endothelial factors and NT-proBNP with parameters of cardiopulmonary exercise testing.

	peak VO_2_ in ml/min/kg			EQCO2			EQO2			Oxygen pulse in ml/beat		
	< cut off	> cut off	p	< cut off	> cut off	p	< cut off	> cut off	p	< cut off	> cut off	p
**Ang-2 (ng/ml)**	6.6±7.3	4.0±4.4	0.23	3.7±3.9	17.7±8.6	0.11	3.4±3.5	7.5±7.5	**0.04**	7.2±7.5	3.2±2.9	**0.03**
**NT-proBNP (pg/ml)**	670±1159	203±371	0.16	229±412	1903±2098	0.30	186±315	612±1079	0.12	608±1020	151±209	0.05
**Ang-1 (ng/ml)**	5.1±2.9	6.2±4.6	0.41	5.8±3.7	11.1±12.3	0.53	5.7±3.3	6.9±6.5	0.47	7.0±5.0	5.5±3.9	0.21
**Tie2 (ng/ml)**	21.9±5.4	20.8±4.1	0.43	20.7±3.9	24.6±10.4	0.58	20.5±4.0	22.5±5.1	0.10	22.5±5.3	20.2±3.7	0.08
**VEGF (pg/ml)**	91±54	122±84	0.21	115±81	161±69	0.34	110±87	133±52	0.32	106±51	123±91	0.36

### Severely Limited Exercise Capacity

There were 16 patients with severely limited exercise capacity in our cohort of 65 patients with cardiopulmonary exercise testing (defined as peakVO_2_<20 ml/min/kg or an EQCO_2_>34 or a combination of both). The areas under the receiver-operating characteristic (ROC) curves for identifying patients with severely limited cardiopulmonary exercise capacity were 0.784 for NT-proBNP and 0.656 for Ang-2.

### Ventricular Physiology

**Figure 3:**
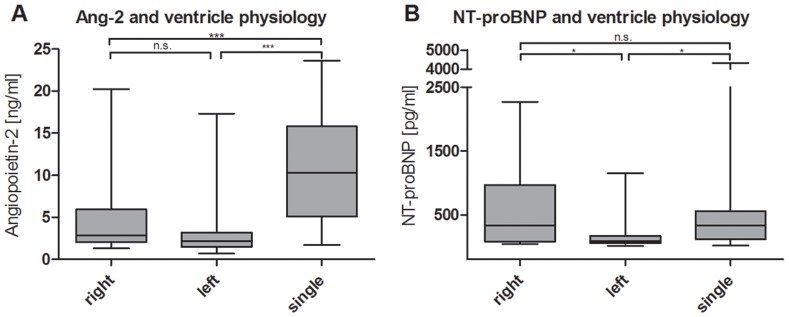
Levels of biomarkers according to ventricular physiology. Ang-2 (A) and NT-proBNP (B) (*p<0.05, **p<0.001, ***p<0.0001).

Ang-2 was significantly elevated in patients with a single ventricle physiology (11.21±6.94 ng/ml) in comparison with patients with a systemic left (2.93±2.75 ng/ml, p<0.0001) or right ventricle (4.86±5.22 ng/ml, p<0.0001), but not between the last two. (**Table 3** and **Figure 3A**) NT-proBNP was elevated in patients with a single ventricle (617±1091 pg/ml) in comparison with a systemic left ventricle (180±250 pg/ml, p<0.001) but not with a systemic right ventricle (642±758 pg/ml). (**Table 3** and **Figure 3B**).

## Discussion

This is the first study to evaluate the role of circulating endothelial factors in adults with congenital heart disease. Ang-2 correlated with parameters of heart failure like NYHA classes and ventricular function. Furthermore, there was a good correlation between Ang-2 and parameters of cardiopulmonary exercise testing. Interestingly, elevated Ang-2 levels were found in patients with a single ventricle physiology.

Two recent studies elucidated the role of circulating endothelial factors in CHF due to acquired heart disease. Both showed an increase of circulating Ang-2 in patients with CHF [21],[22]. Furthermore, Eleuteri et al. reported a stepwise increase of Ang-2 in CHF with increasing NYHA class. We also found elevated Ang-2 levels with increasing NYHA class in our patients. This is of importance, since Norozi et al. showed that the risk of heart failure increases with NYHA class in ACHD [3]. They found an odds ratio for patients in NYHA II compared to patients in NYHA I of 3.4 and for patients in NYHA III of 11.6 [3]. Therefore, Ang-2 might act as a surrogate marker for heart failure in ACHD.

In our study population there was no difference in Ang-1 levels according to NYHA class or ventricular function. This is in accordance with recent studies that reported stable Ang-1 levels in NYHA class I-IV with a trend to lower values in NYHA class III in CHF patients [21],[22]. (**Table 3**).

We observed an increase of Ang-2 levels with worsening ventricular function. There was also a good correlation between ventricular function and NT-proBNP concentrations. This is in contrast to the finding of Larsson and colleagues [4]. In their study, the association of BNP/NT-proBNP with ventricular function was weak and only statistically significant when BNP and NT-proBNP data were combined [4]. It appeared that BNP/NT-proBNP had especially poor value in differentiating between patients with no or mild ventricular impairment, which suggested a limited ability of BNP/NT-proBNP to diagnose heart failure at the initial stages [4].

In patients with CHF an association of elevated circulating Ang-2 and parameters of impaired exercise capacity was recently reported. In 87 patients with heart failure of non-congenital origin circulating Ang-2 levels were associated with lower peak oxygen consumption (peak VO_2_), increased VE/VCO_2_ slope and shorter exercise duration [22]. This is in accordance with our results that a limited cardiopulmonary exercise capacity in ACHD patients is associated with elevated Ang-2 levels. Further, Ang-2 was able to distinguish patients with an especially limited exercise capacity demonstrated by lower peak VO_2_ and increased EQCO_2_. It has been shown that poor exercise capacity identifies ACHD at risk for hospitalization or death [2]. Peak VO_2_ predicted hospitalization or death and was related to the frequency and duration of hospitalization in a large cohort of ACHD [2].

Furthermore, Ang-2 levels were elevated in patients with a single ventricle physiology. This is an interesting finding, because NT-proBNP might have its limitations in this subgroup. It was not elevated in patients with a single ventricle physiology in our study and also showed mixed results regarding its association with a variety of different parameters of heart failure in this population in a recent systematic review [9]. NT-proBNP and BNP are released from cardiomyocytes in response to increased myocardial wall stress due to volume- or pressure-overload states [27]. But in adults with congenital heart disease increased myocardial wall stress due to volume- or pressure-overload states is often not the main pathophysiologic mechanism of heart failure. This is especially true for patients with a single ventricle after the Fontan palliation in whom the leading problem is a limitation of preload [28]. This might explain the restrictions of NT-proBNP as a biomarker for heart failure in this patient group. Further, in patients who underwent the Fontan palliation elevated pulmonary vascular resistance can be found [28]. It was already demonstrated that Ang-2 is elevated and a promising biomarker in patients with elevated pulmonary vascular resistance in the context of idiopathic pulmonary arterial hypertension [29]. Therefore, one possible explanation for the elevated Ang-2 levels in patients with a Fontan circulation could be that elevated pulmonary vascular resistance leads to elevated Ang-2 levels in this subgroup. Interestingly, one study showed that BNP is only elevated in patients with a single ventricle physiology when the systemic ventricle fails but not when there is a failure of the Fontan connection [30]. Thus, Ang-2 may be a valuable biomarker for failure of the Fontan connection in this patient group.

The reason of elevated VEGF and Ang-2 levels in CHF and ACHD remain unclear, because higher levels of these factors have not been translated into clinically significant new vessel formation, as in cancer growth for example [31]. An increase of these angiogenic factors is amongst others possibly triggered by tissue hypoxia [21]. It is hypothesised that elevation of Ang-2 and VEGF in heart failure promotes endothelial repair mechanisms but does not lead to angiogenesis [31],[32]. Although there is a proangiogenic milieu with elevated Ang-2 and VEGF additional endothelial nitric oxidase synthase (eNOS) is needed for the formation of new blood vessels [31]. ENOS is the downstream mediator for VEGF and is lacking in CHF. Current data of our group shows significantly elevated levels of ADMA, the most potent inhibitor of eNOS, in patients with ACHD and heart failure [14]. This could explain why despite elevated angiogenic factors their presence may not necessarily translate into angiogenesis. Therefore, these abnormal levels of angiogenic factors in ACHD may simply play a role in repair and maintenance of a dysfunctional or damaged endothelium [31]. However endothelial repair mechanisms involved remain to be determined.

A limitation of our study is its cross sectional design. A longitudinal design would be needed to elucidate the predictive value of circulating angiogenic factors in patients with ACHD. A long-term follow up study of the patients that participated in this study is planned. This would be of great interest since previous studies suggest that the predictive value of NT-proBNP may be limited in patients with heart failure in ACHD and its true prognostic value is unclear [4],[9] Further, the number of patients enrolled especially those with a single ventricle was small. Therefore, our results have a hypothesis-generating character.

In conclusion, Ang2 could have potential as a biomarker of heart failure in ACHD. It shows especially promising results for patients with a Fontan circulation. A prospective study with a larger patient size is warranted to evaluate the diagnostic and prognostic potential of Ang-2 in this patient group.
